# Training Radiology Residents in Teaching Skills: Using the Kern Curriculum Model to Develop a Pilot, Residents-as-Teachers Curriculum

**DOI:** 10.7759/cureus.104629

**Published:** 2026-03-03

**Authors:** Caroline R Paul, Jeffrey Alpert, Alexander El-Ali, Monica Sheth, Jeffrey Wilhite, Nancy Fefferman

**Affiliations:** 1 Pediatrics, University of Kentucky College of Medicine, Bowling Green, USA; 2 Radiology, Weill Cornell Medicine, New York, USA; 3 Radiology, NYU Grossman School of Medicine, NYU Langone Health, New York, USA; 4 Program for Medical Education Innovations and Research, NYU Grossman School of Medicine, NYU Langone Health, New York, USA

**Keywords:** medical educator, medical resident education, medical teacher, resident as educator, : resident as teacher, resident as teacher curriculum, resident as teacher training, teaching and training residents and medical students

## Abstract

Background and objective

Governing boards require residents to undergo Resident-as-Teacher (RAT) training. Both Radiology residents (RRs) and program directors have reported the need for this training. However, existing literature on radiology curricula remains limited, and information on outcomes, innovative strategies, and feasibility is unclear. In light of this, we aimed to develop, implement, and evaluate a pilot RAT curriculum specific to radiology, based on Kern's curriculum model.

Methods

Based on the Kern model, a curriculum was developed and implemented in 2021-22 and evaluated using electronic pre- and post-module surveys assessing participating residents’ self-reported knowledge, attitudes, and skills. Pre- and post-intervention results were reported as mean ± standard deviation (SD), and differences were analyzed using the Wilcoxon signed-rank test.

Results

Ten residents participated in the Teaching at the Workstation module, with 100% completing pre-and post-evaluation surveys. Residents felt more comfortable addressing gaps in medical students' (MS) skills (pre-test/post-test: 2.6 ± 0.52/3.1 ± 0.57, p<0.025), in understanding best-practice teaching (2.3 ± 0.48/3.1 ± 0.32, p<0.005) and more confident in teaching MS (2.6 ± 0.52/3.2 ± 0.42, p<0.034) and junior residents (2.6 ± 0.52/3.1 ± 0.32, p<0.025). Nine residents participated in the Delivering Effective Feedback module, with 56% completing pre- and post-evaluations. Residents’ understanding of best-practice principles in delivering effective feedback improved, but not significantly (MS: 2.2 ± 0.45/3.3 ± 0.49, p=0.059. Nine residents participated in the Delivering a Case Presentation module, with 100% completing pre-and post-evaluations. They expressed increased comfort and confidence in developing (2.11 ± 0.33/2.91 ± 0.30, p=0.008), preparing (2.11 ± 0.60/2.91 ± 0.30, p=0.020), and delivering presentations (2.22 ± 0.66/3.82 ± 0.40, p=0.025).

Conclusions

A pilot radiology-specific RAT curriculum was successfully developed and implemented. Participating residents reported improvements across multiple knowledge, attitude, and skill domains. By using universal learning strategies and peer-reviewed content, the Kern-based curriculum is potentially generalizable to other specialties. Next steps include incorporating faculty development to support these curricula and assessing higher-level evaluation outcomes.

## Introduction

Governing boards require residents to participate in Resident-as-Teacher (RAT) training regardless of their career path or specialty [1,2]. Furthermore, residents often serve as primary educators for medical students (MS), a responsibility that can occupy up to 25% of their time [3,4,5]. A shortage of dedicated teaching faculty has also reportedly increased the teaching burden on senior residents [4]. Recognizing this issue, medical organizations have disseminated general resources to help residents improve their teaching of medical students [6,7].

Residents have expressed a need for RAT training in radiology and other specialties [8,9]. Radiology program directors have also supported the implementation of RAT training [10]. A recent needs assessment involving both radiology teaching faculty and residents further highlighted the demand for radiology-specific RAT programs [8]. However, to our knowledge, only one published radiology RAT curriculum exists [8,10]. This curriculum consisted of a 1.5-hour seminar for chief residents at the 2010 Association of University Radiologists meeting. Consequently, educators seeking to develop RAT curricula in radiology have limited guidance in the literature regarding achievable objectives, effective teaching strategies, required resources, and measurable outcomes [8,10].

Appropriate curricula should respect the learner’s time, be rigorous in content development, and aim to achieve clearly defined learner objectives. Developing such curricula in medical education can be challenging for several reasons, including the demands of a busy training environment and the often-limited grounding in educational theory and pedagogy that accompanies many well-intentioned teaching efforts. Kern’s curriculum model is a widely recognized framework for medical education curriculum development [11]. It has been successfully applied as the pedagogical foundation for educators who have developed, implemented, and evaluated curricula across various specialties [12,13,14]. Kern’s model comprises six steps: problem identification, local needs assessment, goal and objective formulation, learning strategies, implementation, and evaluation. It is iterative, allowing curriculum developers to revisit earlier steps to strengthen and refine prior implementations of the curriculum.

Given the existing gaps in the literature and the need for robust curricula in radiology training programs, we aimed to develop, implement, and evaluate a radiology RAT curriculum using the Kern model of curriculum design. In this study, we engage in an initial discussion of the curriculum development process, followed by a description of its implementation, execution, and outcomes.

## Materials and methods

The study was conducted in accordance with Health Insurance Portability and Accountability Act (HIPAA) regulations and received approval from our Institutional Review Board (IRB). Consent to use curriculum data was obtained through each radiology resident’s (RR) participation in an IRB-approved resident research registry (#i06-683), which was completed during first-year resident orientation.

Curriculum development and study model

In 2021, a RAT curriculum was developed for RRs following Kern’s model (Table 1).

**Table 1:** Radiology RAT curriculum adapted according to the Kern model RAT: Resident-as-Teacher

Kern’s steps	Radiology RAT curriculum
Step 1: Problem identification and general needs assessment	Literature review
	Historic review of medical students’ evaluations of residents
	Key stakeholders’ input
Step 2: Targeted needs assessment	Surveys of radiology residents and radiology teaching attendings regarding their perceptions of Radiology RAT training
	Identification of learning topics:
	Teaching at the Workstation
	Delivering Effective Feedback
	Creating and Delivering a Radiology Teaching Presentation
Step 3: Goal and objectives	Goal: The resident will develop the skills and knowledge needed for a resident educator to teach medical students and other residents
	Objectives developed for 3 learning modules
	Module 1:Teaching at the Workstation
	Describe key principles of workstation teaching
	Demonstrate key principles of workstation teaching
	Improve confidence in teaching at the workstation
	Module 2: Delivering Effective Feedback
	Describe the value of effective feedback
	Identify qualities of effective feedback
	Describe strategies to deliver effective feedback
	Module 3: Creating and Delivering a Radiology Teaching Presentation
	Describe hallmarks of an effective case to present and the benefits of a thematic case presentation
	List effective ways to use the font
	Understand how to properly prepare key images for presentation
Step 4: Educational strategies	Modules developed to deliver identified learning topics
	Specific learning strategies included:
	Didactics
	Large group debriefs
	Role-playing
Step 5: Implementation	Radiology teaching core faculty group
	The curriculum was implemented in 2021-2022 during the residents' routine work week
	Live sessions
	Modules could be adapted to a virtual format
	Implementation did not require additional time for the resident
Step 6: Assessment, evaluation, and feedback	Pre-intervention and post-intervention evaluation instrument
	Paired analysis to ensure the rigor of data analysis

Following a problem identification (Kern’s Step 1) and a local needs assessment of both RRs and faculty (Kern’s Step 2), learning objectives and learning strategies were developed (Kern’s Steps 3 and 4).

Participants

Target learners included second and third-year RRs from NYU Grossman School of Medicine, NYU Langone Health, a large urban diagnostic radiology (DR) residency program, and an interventional radiology (IR)-Integrated residency program consisting of 44 residents (34 DR and 10 IR-Integrated). These two cohorts were selected because they are heavily involved in the future teaching of medical students and junior residents and have greater availability during their non-clinical training periods.

Implementation

The curriculum, consisting of three modules, was implemented in 2021-22 during the residents’ routine workweek (Kern’s Step 5).

Evaluation

Pre- and post-intervention evaluation surveys focusing on reported knowledge, skills, and attitudes were developed to evaluate gains before and after exposure to each curriculum module (Kern’s Step 6). These evaluation instruments included a series of Likert-style and open-ended questions. Some questions on broad education areas were given to the participants in all three modules. Using Messick’s framework for educational assessment validity, the surveys demonstrated evidence of content validity, with items being derived from input of content experts, learning process experts, education leaders (including clerkship and residency directors), and the vice-chair of education and peer-reviewed curricula in the literature. The surveys were then pilot tested and underwent a “think-out-loud” process to gather evidence for response process validity before implementing them with our trainees [15]. Data were captured and maintained using licensed Qualtrics software (Seattle, WA). Scores were summarized using mean and standard deviation (SD). Paired analyses were performed on pre- and post-intervention scores at each time point using the Wilcoxon signed-rank to explore changes following participation.

## Results

Kern’s Step 1: problem identification

Problem Identification involved a literature review, findings of which demonstrated gaps in the literature in radiology RAT curricula, a historical review of MS evaluations of RRs, and key stakeholders’ input [8]. These sources identified the need for formal curricula to develop Radiology residents as teachers.

Kern's Step 2: local needs assessment

We performed a local needs assessment where RRs and faculty were surveyed regarding their perceptions of radiology RAT training. The needs assessment demonstrated that 82% of RRs and 93% of faculty desired radiology RAT training, respectively. It also identified key learning topics, some of which were congruent with those described in the literature, while others were unique to radiology. Among these topics were Teaching at the Workstation, Delivering Effective Feedback, and Creating and Delivering a Radiology Teaching Presentation [8].

Kern’s Step 3: goal and learning objectives

Objectives were developed for each learning topic during the third step, and all are presented in Table 1. Key objectives were determined through input from content and education experts. The content was designed to reflect general RAT principles reported in the literature across multiple specialties, including radiology. Content experts comprised both radiology and non-radiology educator leaders. For specific radiology content, radiology faculty with experience and skill in direct teaching contributed to the formation of objectives. Process experts were medical educators formally trained in curriculum design and outcome assessment. This combination of radiology and non-radiology expertise enhanced the development of objectives specific to radiology.

Kern’s Step 4: educational strategies

Modules, ranging from one to two hours in duration, were developed as the educational interventions to cover the identified learning topics. This duration was compatible with the residents’ routine weekly didactic sessions. Specific learning strategies were deliberately chosen to present each module’s content. For example, each module included short didactic sessions and large group debriefs with peer-to-peer facilitation, promoting engagement and active learning. Active learning strategies, such as role-plays, were used in modules 1 and 2 to provide residents with practice in preceptor teaching and feedback delivery.

Kern’s Step 5: implementation of education strategies

Faculty facilitators were part of a core radiology teaching group within the Department of Radiology at NYU Grossman School of Medicine, NYU Langone Health. The curriculum modules were rolled out from 2021 to 2022 during the residents’ routine workweek. The modules took place during their didactic core sessions. Faculty and chief residents scheduled this curriculum as part of the annual plan for these sessions. Learning principles were based on peer-reviewed evidence and expert guidance. Details of the modules are as follows:

Module 1: Teaching at the Workstation

The first module was based on Tan’s description of a five-step skills pedagogy as applied to the radiology workstation [16]. Learning objectives are listed in Table 1. Tan based this approach on a well-recognized precepting model [17,18]. It utilized a skills checklist, which was subsequently adapted for Radiology [18]. Steps in this skills checklist included obtaining a commitment regarding the imaging finding, probing for supporting evidence, teaching general rules, and reinforcing correct actions while correcting mistakes. Faculty teaching this module created a worksheet incorporating this checklist, which was used during the session.

The faculty used pediatric and breast imaging cases as examples during the interactive session. After a didactic session covering the steps of the checklist and demonstrating its use, RRs were given the opportunity to practice applying the checklist in a role-playing exercise with other residents. These practice role-plays were facilitated by faculty and peers, who provided real-time feedback. The session concluded with a group debrief to engage the residents and collect broader feedback from all participants. The module was implemented in July 2021.

Module 2: Delivering Effective Feedback

This module was based on well-recognized literature and peer-reviewed content across specialties [19,20]. The module’s learning objectives are listed in Table 1. The module included a didactic presentation on the principles of delivering effective feedback, providing residents with strategies for giving feedback. The session also featured a demonstration of feedback delivery and educator-facilitated peer practice opportunities. A large group discussion at the end of the training highlighted the value of feedback and summarized insights from the group exercises. The module was implemented in July 2022.

Module 3: Delivering a Radiology PowerPoint Case-Based Presentation

The third module featured a didactic session covering principles of giving an effective case presentation as well as key guidelines for a radiology-specific PowerPoint presentation [21,22,23]. Learning objectives are listed in Table 1. Content was drawn from recent peer-reviewed resources, and faculty leading the session also served as content experts for this topic. Teaching materials included lists of “do and don’t” items and “mistake slides” to stimulate discussion during the didactic session. For example, a “bad” slide was first presented to engage learners and later used to illustrate key teaching points. The module was implemented in October 2022.

Kern’s Step 6: evaluation of the curriculum

Results of pre- and post-intervention evaluation measures for modules 1, 2, and 3 are presented in Table 2.

**Table 2:** Mean scores by item with gains and significance SD: standard deviation

	Indicate how much you agree or disagree with each of the following statements	Pre-assessment, mean (SD)	Post-assessment, mean (SD)	Gain (p-value)
Module 1 (N=10)	I know how to identify gaps in knowledge with medical students	2.9 (0.738)	3.2 (0.422)	+0.3 (0.083)
	I know how to address gaps in medical students’ skills	2.6 (0.516)	3.1 (0.568)	+0.5 (0.025)
	I provide effective teaching to junior residents	2.8 (0.422)	3.0 (0.000)	+0.2 (0.157)
	I understand the best-practice principles in teaching effectively at the workstation	2.3 (0.483)	3.1 (0.316)	+0.8 (0.005)
	I feel confident in my teaching skills at the workstation with medical students	2.6 (0.516)	3.2 (0.422)	+0.6 (0.034)
	I feel confident in my teaching skills at the workstation with junior residents	2.6 (0.516)	3.1 (0.316)	+0.5 (0.025)
Module 2 (N=5)	I know how to identify gaps in knowledge with medical students	3.0 (0.707)	3.1 (0.378)	+0.1 (0.317)
	I know how to address gaps in medical students’ skills	2.4 (0.548)	3.1 (0.378)	+0.7 (0.102)
	I provide effective teaching to junior residents	2.8 (0.447)	3.1 (.378)	+0.3 (0.157)
	I understand the best-practice principles in delivering effective feedback to medical students	2.2 (0.447)	3.2 (0.488)	+1.0 (0.059)
	I understand the best-practice principles in delivering effective feedback to junior residents	2.2 (0.447)	3.2 (0.488)	+1.0 (0.059)
Module 3 (N=9)	I know how to identify gaps in knowledge with medical students	2.5 (0.527)	2.6 (0.505)	+0.1 (0.317)
	I know how to address gaps in medical students’ skills	2.7 (0.441)	2.7 (0.437)	+0.0 (1)
	I provide effective teaching to junior residents	2.5 (0.527)	2.5 (0.934)	+0.0 (0.317)
	I would like to learn how to deliver a radiology-specific case presentation to other residents	3.3 (0.500)	3.4 (0.522)	+0.1 (1)
	I would like to learn how to deliver a radiology-specific case presentation to medical students	3.2 (0.441)	3.4 (0.522)	+0.2 (0.317)
	I understand the principles in developing a radiology-specific case presentation for residents	2.1 (0.333)	2.9 (0.302)	+0.8 (0.008)
	I feel confident in my skills in developing a radiology-specific, effective case presentation for other residents	2.1 (0.601)	2.9 (0.302)	+0.8 (0.020)
	I feel confident in my skills in developing a radiology-specific, effective case presentation for medical students	2.4 (0.527)	2.8 (0.405)	+0.4 (0.046)
	I feel confident in my skills in delivering a radiology-specific, effective case presentation for other residents	2.2 (0.667)	3.8 (0.405)	+1.6 (0.025)
	I feel confident in my skills in delivering a radiology-specific, effective case presentation for medical students	2.4 (0.527)	3.0 (0.000)	+0.6 (0.046)

Pre-and post-evaluation survey results

Ten RRs participated in the Teaching at the Workstation module, 100% (n=10) of whom completed both pre- and post- assessments (all of whom indicated they are likely to teach in their future career). There were significant gains in self-reported confidence in addressing gaps in MS skills (pre-mean: 2.6, SD 0.52, post-mean: 3.0, SD: 0.57, p<0.025), in their understanding of best practice principles in teaching effectively at the workstation (pre-mean: 2.3, SD: 0.48, post-mean: 3.1, SD: 0.32, p<0.005), and in their confidence in teaching both MSs (pre-mean: 2.6, SD: 0.52, post-mean: 3.2, SD: 0.42, p<0.034) and junior residents (pre-mean: 2.6, SD: 0.52, post-mean: 3.1, SD: 0.32, p<0.025). In the post-module evaluation survey, 100% (n=10) of RRs endorsed that feedback from residents is valuable to MSs, while 100% (n=10) and 90% (n=9) of RRs also reported they were confident in identifying knowledge gaps and skills gaps in MSs, respectively. Of note, 80% (n=8) of RRs desired formal training in becoming a better educator.

Nine RRs participated in the Delivering Effective Feedback module, 56% (n=5) of whom completed both pre- and post- evaluations. There were modest increases in residents’ self-reported understanding of best practice principles in delivering effective feedback to MSs (pre-mean: 2.2, SD: 0.45, post-mean: 3.2, SD: 0.49, p=0.059) and to junior residents (pre-mean: 2.2, SD: 0.45, post-mean: 3.2, SD: 0.49, p=0.059). There were also increases in residents’ self-reported skills in identifying gaps in MS skills (pre-mean: 3.0, SD: 0.71, post-mean: 3.1, SD: 0.38, p=0.32), in addressing those gaps (pre-mean: 2.4, SD: 0.55, post-mean: 3.1, SD: 0.38, p=0.10), and in teaching confidence when teaching junior residents (pre-mean: 2.8, SD: 0.45, post-mean: 3.1, SD: 0.38, p=0.16). All of the RRs who completed the post evaluation survey (n=7/7) endorsed that feedback from residents is valuable to MSs, reported they were confident in identifying knowledge and skills gaps in MSs, desired formal training in becoming a better educator, and reported they are likely to teach in their future career.

Eleven RRs participated in the Delivering Case Presentation module, 82% (n=9) of whom completed both pre- and post-session evaluations. There were significant gains in eight out of 10 areas, including understanding of the principles of developing a case presentation for residents (pre-mean: 2.1, SD: 0.33, post-mean: 2.9, SD: 0.30, p=0.008) and confidence in preparing a presentation for residents (pre-mean: 2.1, SD: 0.60, post-mean: 2.9, SD: 0.30, p=0.02). There were significant increases in confidence in preparing a presentation for MSs (pre-mean: 2.4, SD: 0.53, post-mean: 2.8, SD: 0.40, p=0.046). RRs also felt more comfortable in delivering presentations to both MSs (pre-mean: 2.4, SD: 0.53, post-mean: 3.0, SD: 0.00, p=0.046) and junior residents (pre-mean: 2.2, SD: 0.67, post-mean: 3.8, SD: 0.40, p=0.025).

RRs expressed interest in learning how to deliver a presentation to MSs (pre-mean: 3.2, SD: 0.44, post-mean: 3.4, SD: 0.52, p=0.317) and junior residents (pre-mean: 3.3, SD: 0.50, post-mean: 3.4, SD: 0.52, p=1.00) at both pre- and post-intervention time points; though this interest did not significantly increase after receiving the module. 100% (n=11) of RRs agreed that feedback from residents is valuable to MSs. 64% (n=5) and 73% (n=8) of RRs who completed the post-intervention evaluation survey reported they were confident in identifying knowledge gaps and skills gaps in MSs, respectively, while 100% (n=11) of RRs desired formal training in becoming a better educator. 100% (n=11) of RRs reported they are likely to teach in their future career.

Potential for expansion to the faculty

Participating RRs were asked about expanding the use of the modules to include faculty training. Most RRs indicated that faculty should receive training similar to that delivered to the residents themselves (90%, n=9 for Teaching at the Workstation; 100%, n=5 for Delivering Effective Feedback; and 89%, n=10 for Delivering a Case Presentation).

## Discussion

The need for formal RAT curricula has been well documented and recognized by both learners and faculty [4,7,8]. However, radiology RAT curricula remain limited. We developed and evaluated a radiology RAT curriculum that can be adapted to other radiology residency programs and, potentially, to other medical specialties internationally. Its content principles are grounded in the international literature. Based on Kern’s curriculum model, this curriculum allows for adaptability and generalizability across programs and specialties, ultimately demonstrating the feasibility and usefulness of integration elsewhere. We are not aware of any other radiology RAT curriculum that has been developed, implemented, and evaluated similarly.

Future studies should assess our RAT curriculum using more rigorous outcome measures and include larger sample sizes. In addition, further research is needed to investigate the impact of the "Teaching in the Workstation" module using higher-level outcome measures, such as evaluating residents’ teaching skills as observed by their peers or learners. Finally, future evaluations could consider perspectives from both learners and faculty and track the learning and teaching interests of graduating residents and fellows.

Our use of Kern’s curriculum model for this RAT curriculum ensured rigor, as the Kern model is a well-recognized framework for curriculum development [11,12,13,14]. This model supported the development and integration of content relevant to the specific needs of our radiology trainees. Our local needs assessment led to the creation of a novel radiology-specific teaching topic: Teaching at the Workstation [8]. This topic was not identified in Kern’s Step 1. While many medical educators use Kern’s Step 1 to guide curriculum development, they often overlook Step 2. The Teaching at the Workstation content would have been missed if a local needs assessment had not been conducted as part of curriculum development in Kern’s Step 2. Radiology residency programs need to incorporate Kern’s local needs assessment step to effectively adapt this curriculum to their specific program. Teaching at the Workstation, a key domain of radiology teaching, has not been previously described in the RAT literature. Nehr originally described this pedagogy in family medicine, and Tan later applied it to a radiology-specific pedagogical model [16,17,18]. We expanded this approach by implementing it in a real-time learning environment.

Our study demonstrated that teaching at the workstation can be a formalized and effective process based on sound pedagogy. This module, developed for this study, presents a method that is not content-specific and therefore can be applied to residents in different specialties. This module contributes to the literature by providing a peer-reviewed method of precepting beyond radiology, enhancing the rigor of “live, on-the-spot teaching” and offering both learners and teachers a structured framework to increase knowledge and skills at the workstation and in comparable settings across other specialties.

Delivering Effective Feedback is a common topic in RAT curricula across specialties; however, it has not previously been described in the radiology literature. This study demonstrates the feasibility of a Delivering Effective Feedback module for radiology RAT. In our case, the statistically non-significant gains may be due to challenges in understanding the definition and meaning of feedback [24,25]. It may also be related to the small sample size in this module. Nonetheless, meaningful educational gains were observed, such as evidence that peer-reviewed content and the use of multiple learning strategies in a short, focused module can be adapted for other programs in a broadly applicable manner.

Participants reported the need for faculty development and indicated that faculty should receive the same RAT training that the residents completed in this curriculum. Faculty development in RAT topics would enable instructors to model and demonstrate effective teaching to residents. Such modeling is likely to encourage residents who plan to teach in their future careers.

One limitation of our study is its single-site, single-cohort design, which relied on self-reported gains and changes in knowledge, skills, and attributes such as confidence as quantitative measures of curriculum success. The small number of residents who participated in the modules is also a limitation, but this reflects the realities of residency education, as those who did not attend were on vacation, ill, or limited by clinical and post-call duties. Nonetheless, one of the strengths of our study was that evaluation phases were conducted during real-time learning, further supporting its feasibility for use across other institutions and specialties.

Despite these limitations, our study demonstrated that an innovative, rigorously developed RAT curriculum was feasible, effective, and likely generalizable. The curriculum addressed essential topics recognized in RAT literature and was successfully implemented during the residents’ workweek, avoiding additional time over a one-year academic period. We are not aware of any other radiology RAT curriculum that has been developed, implemented, and evaluated similarly.

## Conclusions

This study describes a successfully implemented radiology RAT curriculum in the radiology residency program at NYU Grossman School of Medicine, NYU Langone Health. The curriculum resulted in self-reported gains for residents in knowledge, skills, and confidence. Grounded in sound pedagogical principles, this curriculum could be utilized in other radiology residency programs in its current form or adapted for use in different educational settings and specialties. It may also serve as a framework for faculty development to advance RAT education.
